# Supplementary material for: Increased Stiffness Downregulates Focal Adhesion Kinase Expression in Pancreatic Cancer Cells Cultured in 3D Self-Assembling Peptide Scaffolds
Source: Biomedicines. 2022 Jul 29;10(8):1835. doi: 10.3390/biomedicines10081835 (PMC9405295; doi:10.3390/biomedicines10081835)
Supplement: Supplementary file 1 [file biomedicines-10-01835-s001.zip › biomedicines-1828406-supplementary.pdf]

Table S1. Approximate stiffness of RAD16-I hydrogels (Pa) depending on peptide concentration, measured by rheometry (Sieminski et al. 2007).

Peptide concentration (%, w/v)	G′ hydrogel (Pa)
0.15	125
0.25	450
0.3	725
0.5	2,500
0.8	8,500
1	15,000

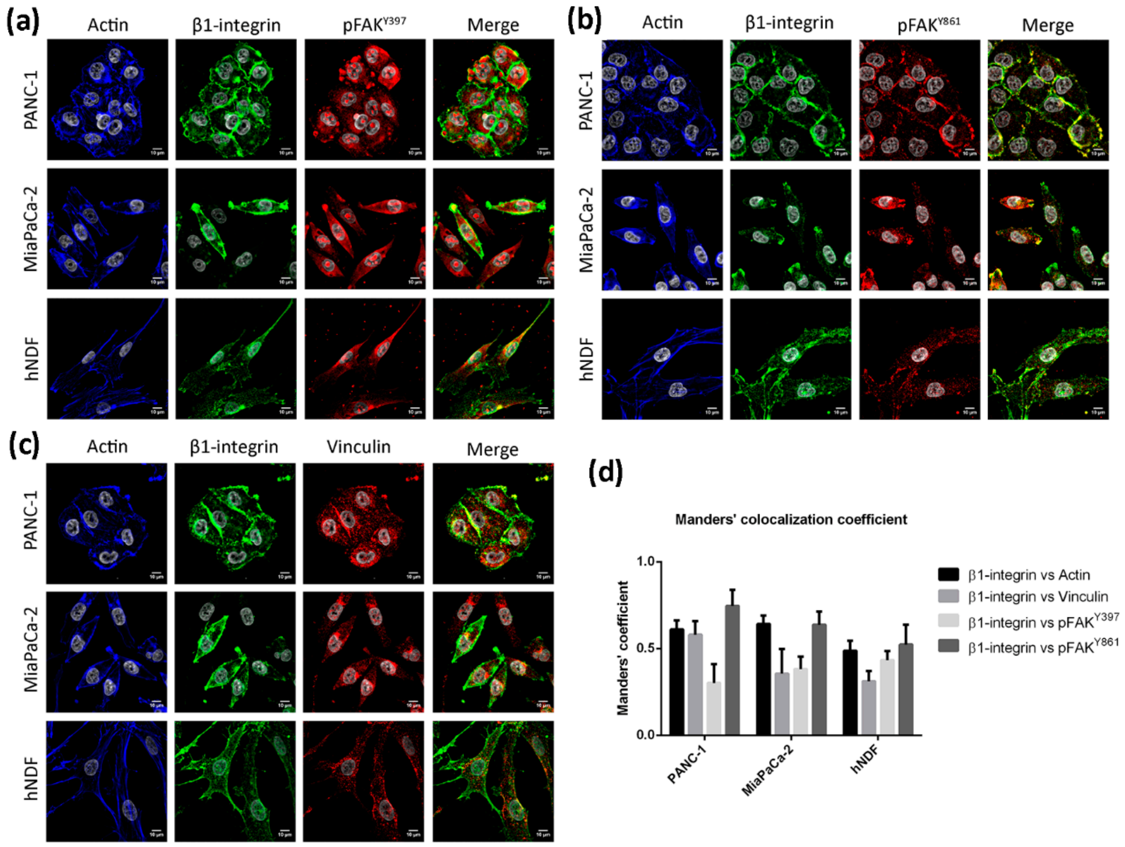

Figure S1. Immunofluorescence analysis of signal mechanotransduction proteins in 2D cultures of PANC-1, MiaPaCa-2 and hNDF cells. **(a)** Actin, β1-integrin and pFAK^{Y397} staining; **(b)** Actin, β1-integrin and pFAK^{Y861} staining; **(c)** Actin, β1-integrin and vinculin staining; **(d)** Manders' colocalization coefficients. Scale bars represent 10 μm.

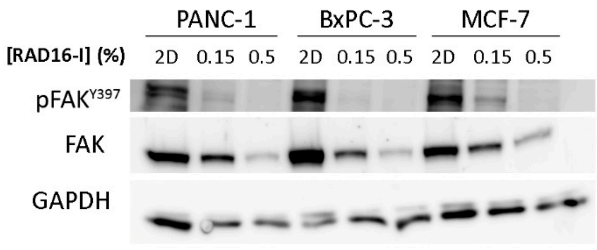

Figure S2. Western blot bands of pFAK^{Y397} and FAK expression in PDAC cell lines (PANC-1 and BxPC-3) and the breast cancer cell line MCF-7 cultured in 2D and in RAD16-I hydrogels at 0.15% and 0.5% peptide concentration. GAPDH was used as loading control.

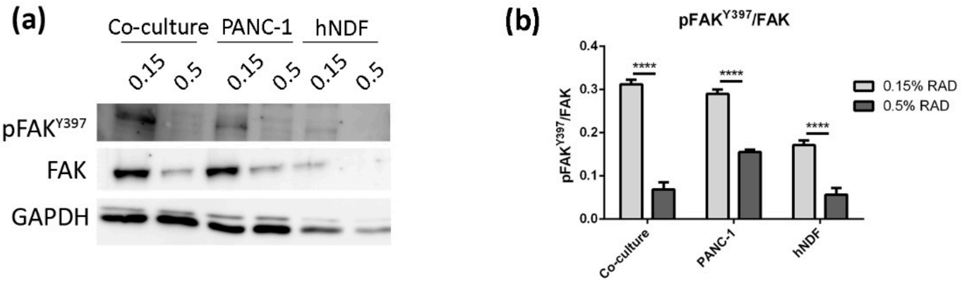

Figure S3. Analysis of FAK activation in RAD16-I 3D co-cultures and mono-cultures of PANC-1 and hNDF. **(a)** Western blot bands of FAK and pFAK^{Y397}; **(b)** Densitometry of bands shown in **(a)**.

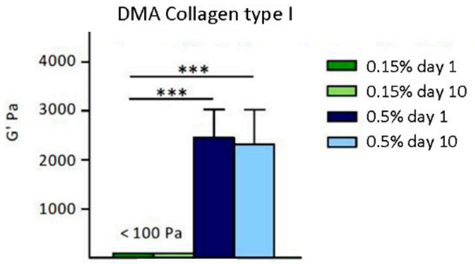

Figure S4. DMA analysis of 0.15% and 0.5% Collagen type I gels at day 1 and 10 of culture with PANC-1 cells.

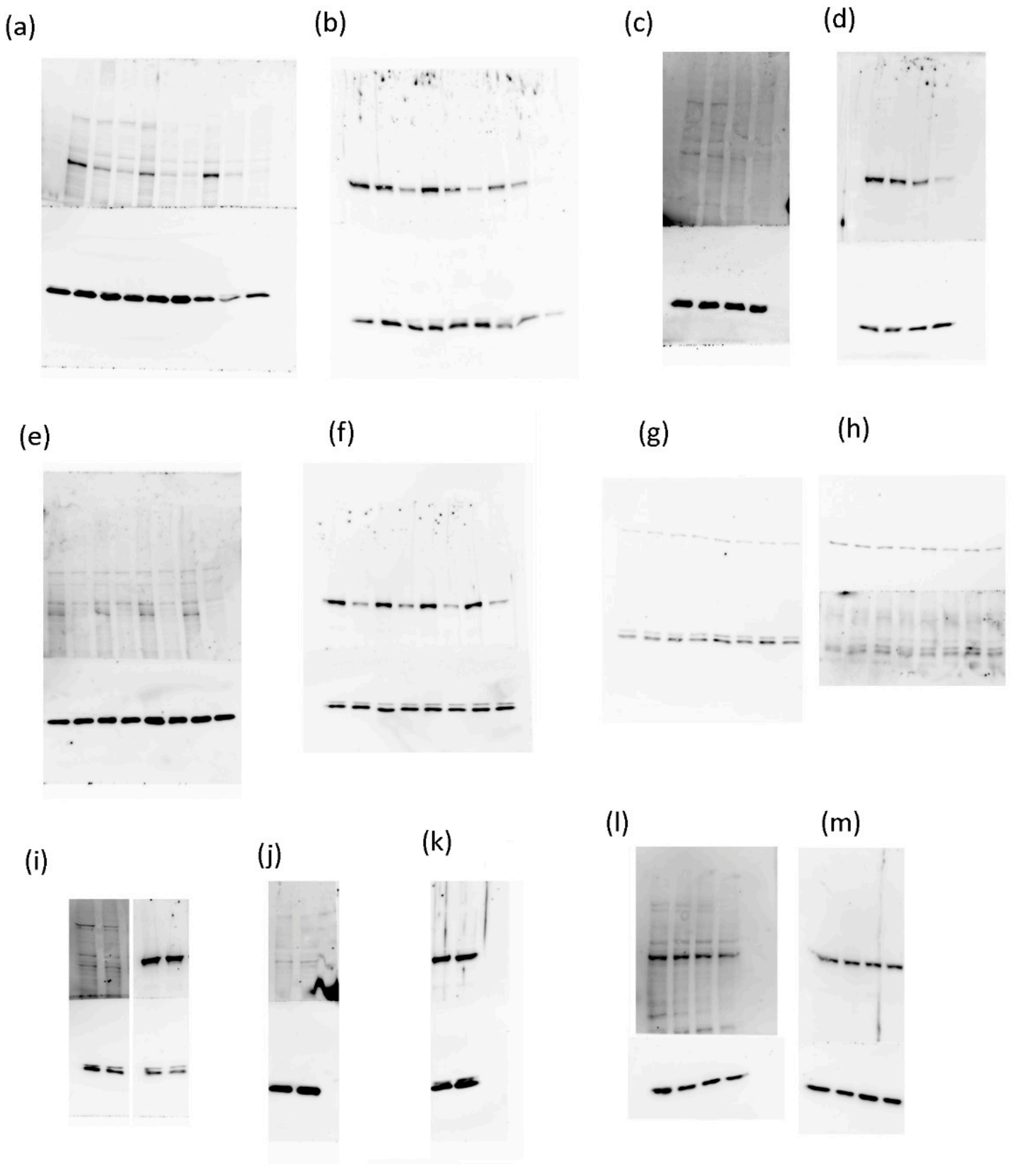

Figure S5. Uncropped western blots. Western blots from Figure 4 (a – d), Figure 5 (e – h), and Figure 6 (i – m).
